# The hidden seasonality of pharyngitis and tonsillitis: a recurring early-summer wave of unclear aetiology

**DOI:** 10.1017/S0950268825100393

**Published:** 2025-08-15

**Authors:** Marcin Piotr Walkowiak, Jarosław Walkowiak, Jarosław Szydłowski, Dariusz Walkowiak

**Affiliations:** 1 Department of Preventive Medicine, Poznan University of Medical Sciences, Poznań, Poland; 2 Department of Pediatric Gastroenterology and Metabolic Diseases, Poznan University of Medical Sciences, Poznań, Poland; 3 Pediatric Otolaryngology Department, Poznan University of Medical Sciences, Poznań, Poland; 4 Department of Organization and Management in Health Care, Poznan University of Medical Sciences, Poznań, Poland

**Keywords:** paediatrics, pharyngitis, seasonal variation, temperature, tonsillitis

## Abstract

Limited studies on the seasonality of pharyngitis and tonsillitis suggest subtle but unexplained fluctuations in case numbers that deviate from patterns seen in other respiratory diagnoses. Data on weekly acute respiratory infection diagnoses from 2010–2022, provided by the Polish National Healthcare Fund, included a total of 360 million visits. Daily mean temperature and relative humidity were sourced from the Copernicus Climate Data Store. Seasonal pattern was estimated using the STL model, while the impact of temperature was calculated with SARIMAX. A recurring early-summer wave of an unspecified pathogen causing pharyngitis and tonsillitis was identified. The strongest pattern was observed in children under 10, though other age groups also showed somewhat elevated case numbers. The reproductive number of the pathogen is modulated by warmer temperatures; however, summer holidays and pandemic restrictions interrupt its spread. The infection wave is relatively flat, suggesting either genuinely slow spread or multiple waves of related pathogens. Symptomatic data unambiguously demonstrate existence of pathogens of quite distinct characteristics. Given its consistent year-to-year pattern, identifying these potential pathogens could enhance respective treatment, including antibiotic therapy.

## Introduction

The annual cycle of respiratory infections in primary care appears straightforward – viruses generally thrive in lower temperatures, leading to peak infection rates during colder months. Based on the preferences of particular strains, infection seasons may vary slightly; for example, high number of milder cases arise in early autumn, while more severe infections tend to peak in early spring, as seen with influenza [1]. Moreover, infections caused by specific pathogens exhibit considerable variability in severity and symptomatology when analysed using symptomatic data. While tonsillitis and pharyngitis generally follow this seasonal pattern of other respiratory diagnoses, closer scrutiny reveals a subtle deviation during late spring and early summer. During this period, diagnoses of other respiratory infections decline, yet cases of tonsillitis and pharyngitis persist, suggesting a unique seasonal aetiology.

Deviations from the overall seasonal pattern of respiratory infections have been documented in prior studies. Lipsett et al. noted that in the United States, pharyngitis exhibits minimal annual fluctuation compared to other respiratory infections [2]. Andersson et al. observed in Sweden a relatively flat cycle with a minor dip in late summer for pharyngotonsillitis [3]. Chen et al. found that in southern China, tonsillitis was the most prevalent in early summer and detected some correlation with temperature [4]. Building on their work, we previously identified an early summer wave of tonsillitis and a smaller wave of pharyngitis among paediatric patients in Poland [1], prompting further exploration of this anomaly.

Some understanding of the progression dynamics of these infections may be inferred indirectly from hospitalization data. The relatively flat seasonal cycle observed in tonsillitis and pharyngitis, distinct from the pronounced peaks of other respiratory infections, could suggest a role for chronic or recurrent tonsillar susceptibility with a merely secondary role of acute viral surges. However, this hypothesis was largely ruled out during the COVID-19 pandemic: Studies across multiple countries independently documented a sustained reduction in tonsillectomies, clearly indicating that the usual pathogens responsible for recurring tonsillitis were indeed suppressed by pandemic restrictions [5, 6]. Chadha observed a subtle bimodal pattern in haemorrhage risk following tonsillectomies in the United Kingdom. While he suspected respiratory infections as a plausible cause, he could not account for the summer peak [7]. Additionally, studies on the seasonality of peritonsillar abscess have clearly shown that this particular condition was somewhat more likely in warmer seasons [8–10].

This study aims to characterize infection waves caused by elusive potential pathogens contributing to the recurrent summer wave of tonsillitis and pharyngitis. By analysing symptomatic data, we aim to find the timing and affected demographics. These findings provide a foundation for more targeted identification efforts and offer a potential basis for seasonally adjusted antibiotic guidelines, improving treatment precision and efficacy.

## Data source

Weekly data on Acute Respiratory Infections (ARI), classified under ICD-10 codes J00–J22, were obtained from the Polish National Healthcare Fund, spanning 2010-W1 to 2022-W52. The dataset, encompassing nearly all primary healthcare visits, included approximately 360 million visits within Poland’s universal healthcare system. To ensure privacy, weekly diagnoses between 1 and 4 were reported as ‘ < 5’ and estimated as 2 for analysis, except for individuals over 100, where the count was assumed to be 1. Rarely used codes (<0.01% of visits) were merged with more common ones; for example, all pneumonia cases, regardless of pathogen identification, were grouped together. Daily mean temperature and relative humidity data were sourced from the Copernicus Climate Data Store (‘E-OBS daily gridded meteorological data for Europe’) to calculate average weekly values for the entire country. Tonsillitis was defined solely by the ICD-10 code J03, and pharyngitis was defined solely by the code J02, serving as the key metrics for analysis.

## Calculation

For visualization purposes, the annual pattern of analysed diagnoses was extracted from the cyclical component of STL (Seasonal-Trend Decomposition using LOESS) with a fixed cycle and presented using splines. To assess whether the summer wave is genuinely temperature-driven or coincidental, it was necessary to use a model that accounts for temporal correlation between observations, allowing for fluctuations in case numbers while controlling for confounding respiratory infections and seasonal factors, such as summer holidays. A Seasonal AutoRegressive Integrated Moving Average with eXogenous variables (SARIMAX) (1,0,1)(1,0,1,52) model was selected, using paediatric tonsillitis as the sole outcome variable, due to its pronounced and distinct epidemic wave, which facilitated robust modelling of seasonal dynamics. This configuration was chosen as the simplest model that leverages both autoregressive and moving average components to capture weekly and seasonal dynamics in paediatric tonsillitis, with all parameters verified to be statistically significant (p < 0.01). Case numbers were log-transformed to ensure that coefficients reflect changes in the pathogen’s effective reproduction number and to ensure stationarity, which was further confirmed with the Ljung–Box test. To adjust for tonsillitis cases caused by common winter viruses, the relative week-to-week change in common cold cases was included as an explanatory variable. Since SARIMAX inherently accounts for seasonality, temperature was expressed as the difference from the corresponding week in the prior year. This approach aimed to determine whether a relatively hotter week, compared to the same week in the previous year, would lead to an increased effective reproduction number for the inferred pathogen, thereby demonstrating the role of temperature even after adjusting for seasonality.

While direct calculation of the inferred pathogen’s reproductive number was not feasible due to the lack of direct detection or data on its incubation period, it could be indirectly calculated based on the increase in specific case numbers over the typical proportion of diagnoses expected from overall patient visits for respiratory infections, enabling consistent comparison with estimates derived using the same method for other pathogens. To calculate the effective weekly reproductive number, the number of specific respiratory cases was aggregated across age groups 1–17 years. To isolate outbreak-related cases, a baseline representing predominant diagnostic proportions was estimated using Non-negative Matrix Factorization (NMF). NMF was applied to the case count matrix with a single component, reconstructed into feature space, and subtracted from the original dataset to detect transient periods with a unique surplus of specific diagnoses over the baseline level expected from the overall number of cases. The surplus was log-transformed to enable calculation of relative increases. The periods relevant for specific diagnoses were narrowed to the period from the local minimum to the local maximum to capture only the growth phase. To calculate the effective reproductive number robustly, the slope was estimated using the non-parametric Theil–Sen estimator, with confidence intervals derived via bootstrapping using 1,000 samples from all valid years.

Data processing and analysis were performed using Python 3.10 with pandas 1.4.3. STL and SARIMAX were calculated using Statsmodels 0.14.0, while NFM using NMF was implemented with sklearn 0.0.post1. Graphs were generated using matplotlib 3.8.0 with splines calculated using scipy 1.9.3, which was moreover also used for the Theil–Sen estimator.

**Figure 1.**
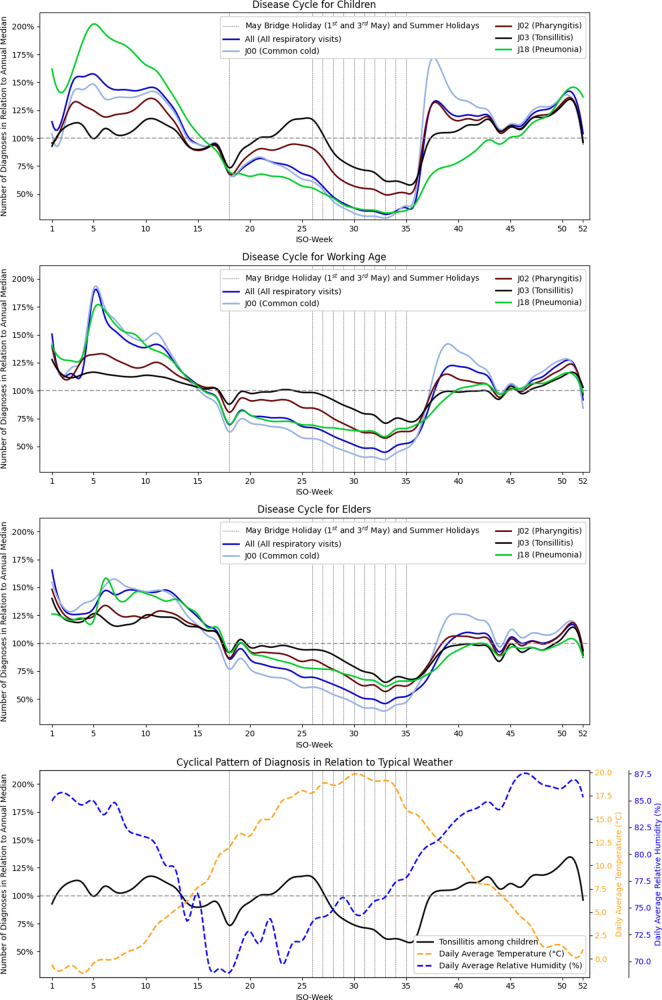
Annual cycle of selected respiratory diagnoses and climatic factors.

The annual cycle of diagnoses, shown in Figure 1, aligns with Poland’s education calendar. Summer school holidays are nationwide and synchronized, with summer breaks spanning July–August, though kindergartens open one month each in a staggered, alternating schedule. School education starts at age 7. The pattern for tonsillitis and pharyngitis is flatter than that of other respiratory infections but largely follows their seasonal trends. For all diagnoses, in all age groups, an annual minimum occurs in late summer, followed by a surge in early autumn, with a pronounced increase during the influenza season at the start of the year. However, starting around ISO week 19, in early May, the pattern becomes divergent. In adults, tonsillitis cases remain nearly flat, even as other respiratory diagnoses show a steady decline. In children, this trend is even more pronounced, with both tonsillitis and pharyngitis cases showing a clear increase that cannot be attributed to lingering infections, but instead indicates a distinct infection wave. The paediatric tonsillitis peak occurs during a relatively warm and dry period of the year; however, beyond meteorological factors, infection numbers sharply decline at the end of the school year, despite rising temperatures.

**Figure 2.**
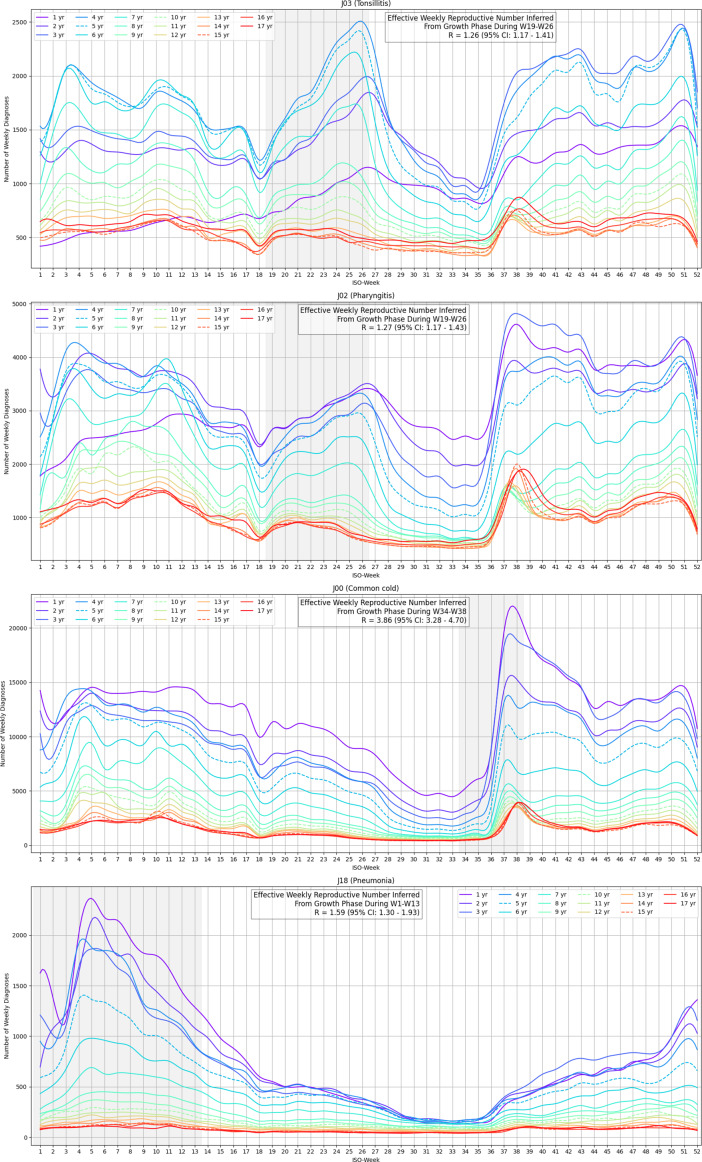
Annual cycle of particular paediatric respiratory diagnoses in age groups.

Paediatric patients are further divided into age cohorts in Figure 2. Summer tonsillitis diagnoses are most prevalent around age 4, with numbers oscillating near the annual high typically observed at the onset of the influenza season. A subtle shift in timing is noted: younger age groups experience the summer wave later. For children over 10, it is less pronounced but remains distinct when compared to other diagnoses in the same age groups. A similar, albeit weaker, pattern is observed in pharyngitis. The shape of the infection wave deviates from the expected SIR model (Susceptible, Infectious, Recovered); the sharp declines suggest a sudden discontinuity in transmission rather than a gradual reduction driven by susceptible depletion.

The summer infection wave exhibits markedly slower growth (weekly effective R = 1.26, 95% CI: 1.17–1.41) compared to other recurrent infection waves, such as the early autumn common cold surge (R = 3.86, 95% CI: 3.25–4.75) or influenza (R = 2.12, 95% CI: 1.68–2.69 for cases officially attributed to J11 influenza, heavily underdiagnosed; R = 1.59, 95% CI: 1.30–1.93 for a low-end estimate based on pneumonia cases during influenza season). An interesting pattern emerges in the autumn data: an initial spike in upper respiratory infections is predominantly diagnosed as common cold, but a moderate share manifests as pharyngitis or tonsillitis. In younger age groups, this is followed by a gradual increase or even a secondary wave of pharyngitis or tonsillitis. This shift in diagnosis proportions coincides with early autumn, when temperature conditions resemble those at the onset of the summer wave, and the affected age groups are similar. This pattern suggests that factors favouring the summer wave, possibly including a persistent pathogen, may also contribute to autumn cases, though rhinovirus prevalence in autumn may obscure this signal.

**Figure 3.**
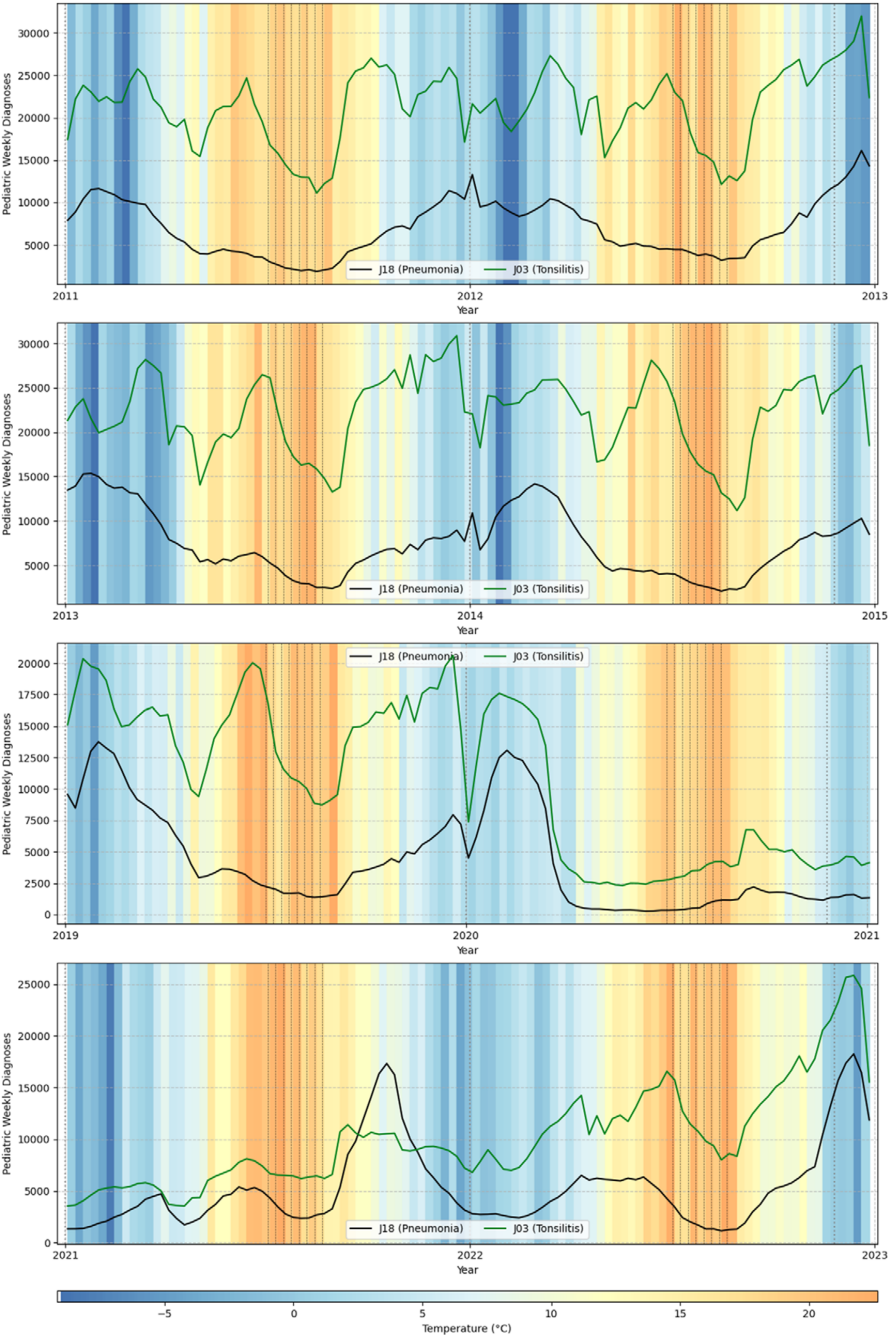
Number of tonsillitis and pneumonia cases in the analysed period in relation to weekly temperature in selected years.

Figure 3 shows the crude number of paediatric tonsillitis cases, with paediatric pneumonia as a reference for overall epidemiological trends. While this pathogen tended to surge in early summer before holidays, it had its peak in late spring, thus showing a weak link to rising temperatures. Tonsillitis cases fell dramatically during the initial COVID-19 lockdown. Although some patients avoided seeking medical care for respiratory symptoms due to concerns about COVID-19 restrictions [11], this alone does not explain the decline, given that pneumonia cases dropped even more sharply. Following an initial decrease, tonsillitis cases began to rise through the summer of 2020. By 2021, a distinct summer tonsillitis peak was evident, though somewhat weaker than in typical years. In contrast, after lifting restrictions, the summer of 2022 appeared typical, unlike the pronounced outbreaks of other pathogens observed at the end of 2022.

The model presented in Table 1 identifies predictors of paediatric tonsillitis. The strongest predictor is the autoregressive component, which is unsurprising, as it essentially indicates that the number of cases in a given week depends on the previous week. The moving average component has a positive sign, suggesting that it does not correct for random noise in weekly data but rather captures short-term trends related to the rise and fall of infection waves. However, in the seasonal component, the roles of the autoregressive and moving average coefficients were more typical. The number of tonsillitis cases is strongly influenced by weekly changes in common cold cases, reflecting respiratory virus trends.

**Table 1.** Paediatric tonsillitis predictors in SARIMAX (1,0,1)(1,0,1,52) model

	Coef.	Std. err.	z	P > |z|	C.I. 0.025	C.I. 0.975
Common cold change.Lag1 (Lag1-in relation to previous week)	0.3374	0.006	55.031	0.000	0.325	0.349
Temperature change.Lag52 (Lag52-in relation to previous year)	0.0024	0.001	3.213	0.001	0.001	0.004
AutoRegressive.Lag1	0.9997	0.000	2,273.205	0.000	0.999	1.001
MovingAverage.Lag1	0.3139	0.025	12.443	0.000	0.264	0.363
AutoRegressive.Seasonal.Lag52	0.9959	0.011	88.195	0.000	0.974	1.018
MovingAverage.Seasonal.Lag52	−0.9393	0.083	−11.371	0.000	−1.101	−0.777
sigma2	0.0078	0.001	14.633	0.000	0.007	0.009

The impact of temperature change relative to the previous year, while the weakest variable, is nonetheless statistically significant. Each 1 °C increase in temperature correlates with a 0.24 percentage point increase in the effective reproduction number of the pathogen causing tonsillitis (C.I. 95% 0.1–0.4). The SARIMAX model showed a small residual variance (sigma2 = 0.0078), expected due to the heterogeneity of infections underlying the diagnosis.

## Discussion

The results clearly demonstrate the existence of an inferred pathogen responsible for a summer infection wave causing tonsillitis and pharyngitis. This infection wave is most pronounced in tonsillitis cases among children under 10 years of age, though elevated diagnoses in other age groups suggest broader susceptibility. The pathogen appears to spread slowly, with an estimated weekly reproductive number significantly lower than that of rhinoviruses or influenza. It exhibits a clear preference for warmer temperatures, as even after controlling for seasonality, the temperature difference relative to the previous year remains a statistically significant predictor. However, infections are curtailed during school closures. Potential identification of this pathogen could allow adjustment of treatment for those conditions based on seasonal factors.

The observed infection seems to attract very limited attention from prior studies as it occurs during a seasonal lull in respiratory infections, with case numbers remaining relatively inconspicuous. Interestingly, related hospitalization data were implicitly showing a very subtle but matching pattern suggesting the existence of some summer infection wave [7], though without determining the responsible pathogens, it would be too early to confirm linkage.

The apparent slow spread of the pathogen could be interpreted in two ways. It may indicate that this particular pathogen genuinely spreads slowly, potentially due to a long incubation or infectious period. Alternatively, it could suggest the presence of several similarly behaving strains, whose overlapping infection waves create the impression of a prolonged infection wave. Given that the data consistently shows a pattern approaching a single summer peak, the interpretation of a genuinely slow spread is favoured. This is further consistent with slow post-COVID rebound and relatively subtle estimated impact of temperature on effective reproduction number – such a weak relation is only sufficient to modulate seasonal wave in case of the impact slowly compounding over longer periods.

Available literature does not clearly identify the pathogen behind the early-summer wave of pharyngitis and tonsillitis, with studies either inconclusive or suggesting common pathogens are unlikely. Group A Streptococcus (GAS), a typical cause, peaks in colder months [12, 13], and its positivity rate in pharyngitis cases drops in summer [3], hinting at another pathogen. *Fusobacterium necrophorum* is inconsistent with the pattern, as it predominantly affects teenagers [14] and showed no major change during lockdowns [6].

Among viruses linked to these conditions, adenovirus could be a candidate due to its frequent asymptomatic detection in removed tonsils [15]. However, it ranks second only to rhinovirus (RHV) in asymptomatic prevalence among healthy controls [16], warranting caution in interpretation. Its seasonality is insufficient, with subtle peaks temporally misaligned [17–19]. Similarly, Epstein–Barr virus (EBV), though less studied, appears to lack sufficient seasonality [20] or outright have misaligned seasonality [21]. Although enteroviruses display summer seasonality that could align with the observed wave, existing studies indicate a broader dispersion than seen in our symptomatic data [22, 23]. Hence, an unidentified pathogen or a unique strain of a known virus, with distinct seasonal and clinical traits, might explain the observed infection wave. Additionally, studies on the impact of COVID-19 interruptions on other respiratory infections demonstrated that rhinoviruses and, to a lesser extent, adenoviruses were the least affected, making them unlikely suspects for modulating the early summer wave [24, 25].

## Conclusion

The analysis indicates a recurring early-summer peak in tonsillitis and pharyngitis, most evident in young children, pointing to an inferred pathogen. The association with warmer temperatures highlights the need for microbiological studies to determine the pathogen’s nature. As those conditions may be treated with an antibiotic, confirming the nature of this pathogen may allow us to take advantage of its seasonality to adjust the treatment guidelines accordingly during only for that particular part of the year, though not only feasibility but direction of this change could not be determined without further studies and microbiological confirmation.

## Limitations

As studies on geographical differences in infection waves show some regional variability [26], the exact timing of this wave in nearby countries may differ due to climatic factors and variations in school holiday schedules. Given the modest inconsistencies in diagnosing respiratory conditions across regions in Poland and the relative homogeneity of the country, the data are insufficiently detailed for spatio-temporal analysis, while the actual shifts in the proportions of symptomatic, medically attended infections may be slightly underestimated due to coding challenges [27].

## Data Availability

Temperature data: https://cds.climate.copernicus.eu/datasets/insitu-gridded-observations-europe?tab=overview. Infection data available from the authors at reasonable request.
